# Supplementary material for: Correlative cellular ptychography with functionalized nanoparticles at the Fe L-edge
Source: Sci Rep. 2017 Jul 6;7:4757. doi: 10.1038/s41598-017-04784-5 (PMC5500580; doi:10.1038/s41598-017-04784-5)
Supplement: Supplementary file 1 — Supplementary Information [file 41598_2017_4784_MOESM1_ESM.pdf]

Supplementary Information

Correlative cellular ptychography with functionalized nanoparticles at the Fe L-edge

Marcus Gallagher-Jones, Carlos Sato Baraldi-Dias, Alan Pryor, Jr., Karim Bouchmella, Lingrong Zhao, Yuan Hung Lo, Mateus Borba Cardoso, David Shapiro, Jose Rodriguez and Jianwei Miao

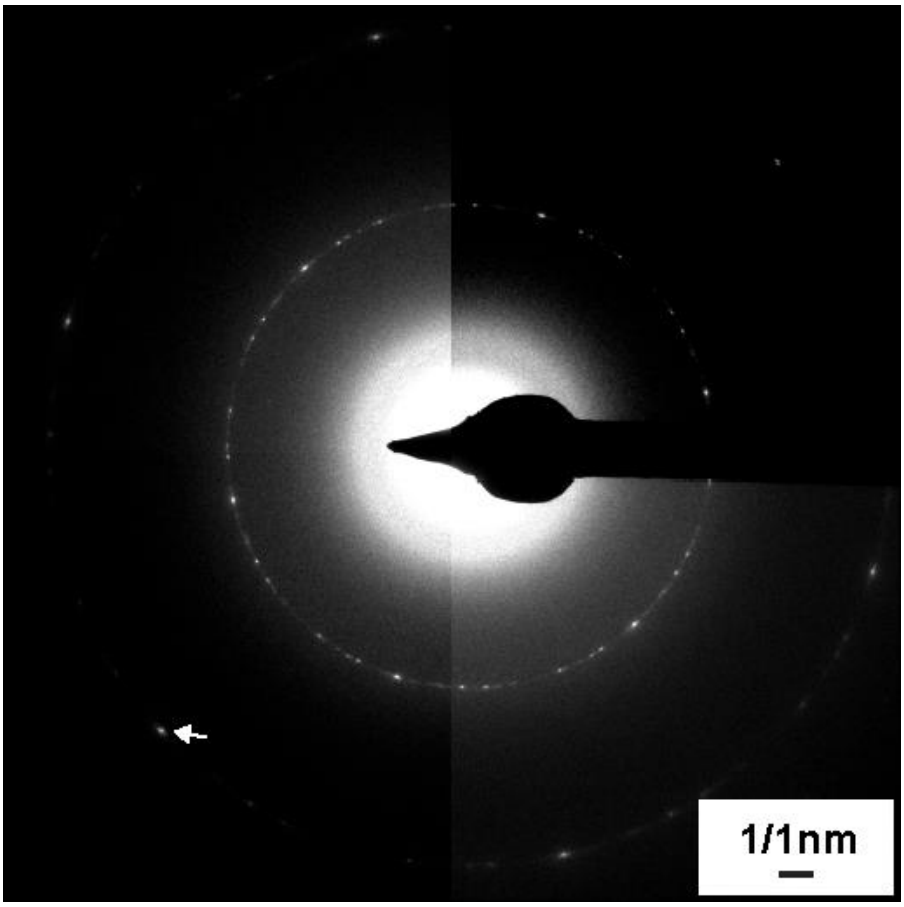

Supplementary Figure 1. Electron diffraction from Au-LaceyGO grids. White arrows point to reflections produced by individual graphene crystals. The different contrast within the image is due to the differential gain of the 4 tiled detector.

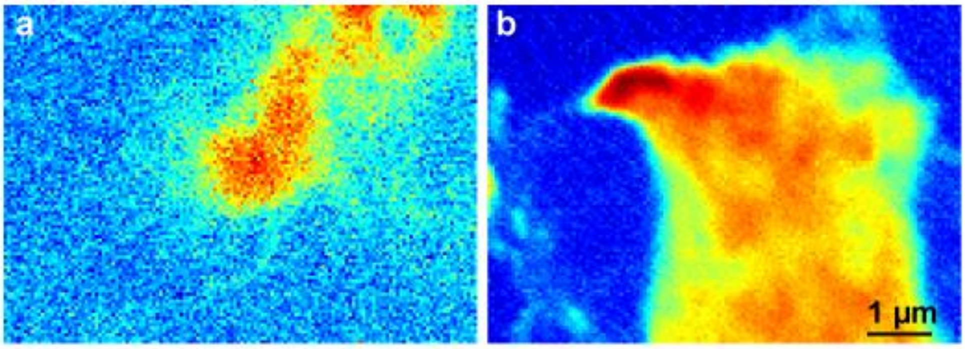

Supplementary Figure 2. Comparison of STXM images on two different substrates. STXM images of the edge of a HeLa cell on (**a**) 50 nm thick Si_3N_4 with a 2 ms dwell time and (**b**) atomically thin graphene oxide with a 3 ms dwell time. The SNR, defined as the quotient of the average intensity inside the sample and the standard deviation of the surrounding region, is approximately 2.9 and 12.1, respectively, which is well beyond the effect of the difference in exposure time. The STXM probe was moved in 50 nm increments with a 100 nm focal spot size.

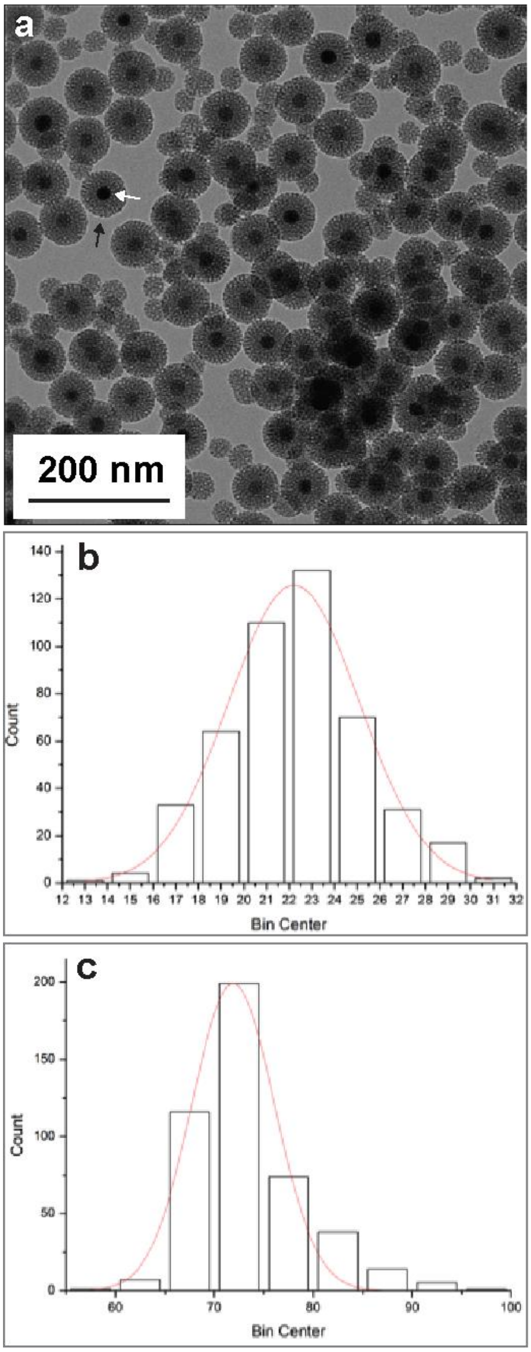

Supplementary Figure 3. Structural analysis of Fe_3O_4/SiO_2 (core-shell) nanoparticles. (**a**) TEM image of core-shell nanoparticles; scale bar, 200 nm. White arrow points to Fe_3O_4 core, black arrow points to SiO_2 shell. (**b**) Quantitative analysis of distribution of Fe_3O_4 core sizes in a nanoparticle preparation; histogram shows a fit based on a normalized distribution with a mean of approximately 22 nm. (**c**) Quantitative analysis of core-shell nanoparticle sizes; histogram shows a fit with a normalized distribution with a mean of approximately 73 nm. In panels b and c, the y-axis shows counts and the x-axis bin centers represent the size in nanometers.

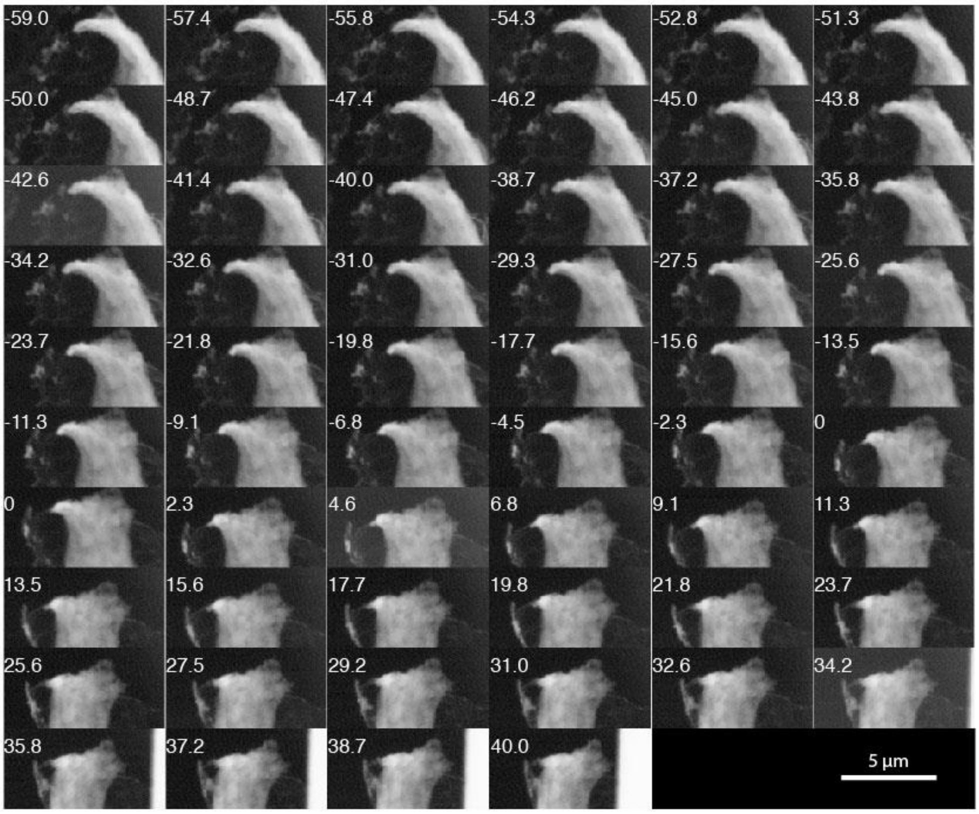

Supplementary Figure 4. STXM tomographic tilt series ranging from -59° to +40° in equal slope increments.

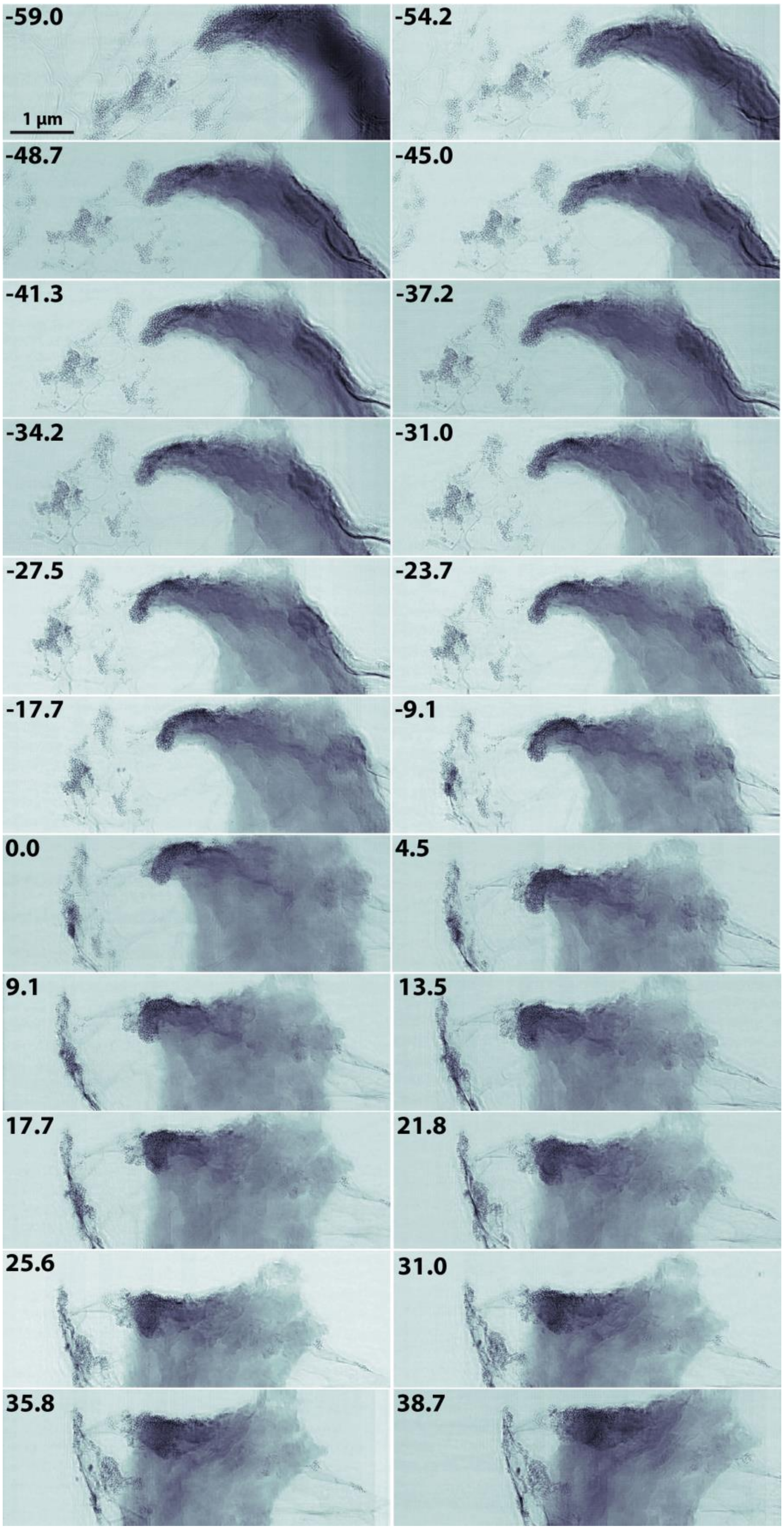

Supplementary Figure 5. Magnitude images of the ptychographic tomography tilt series ranging from -59° to +38.7° in equal slope increments.

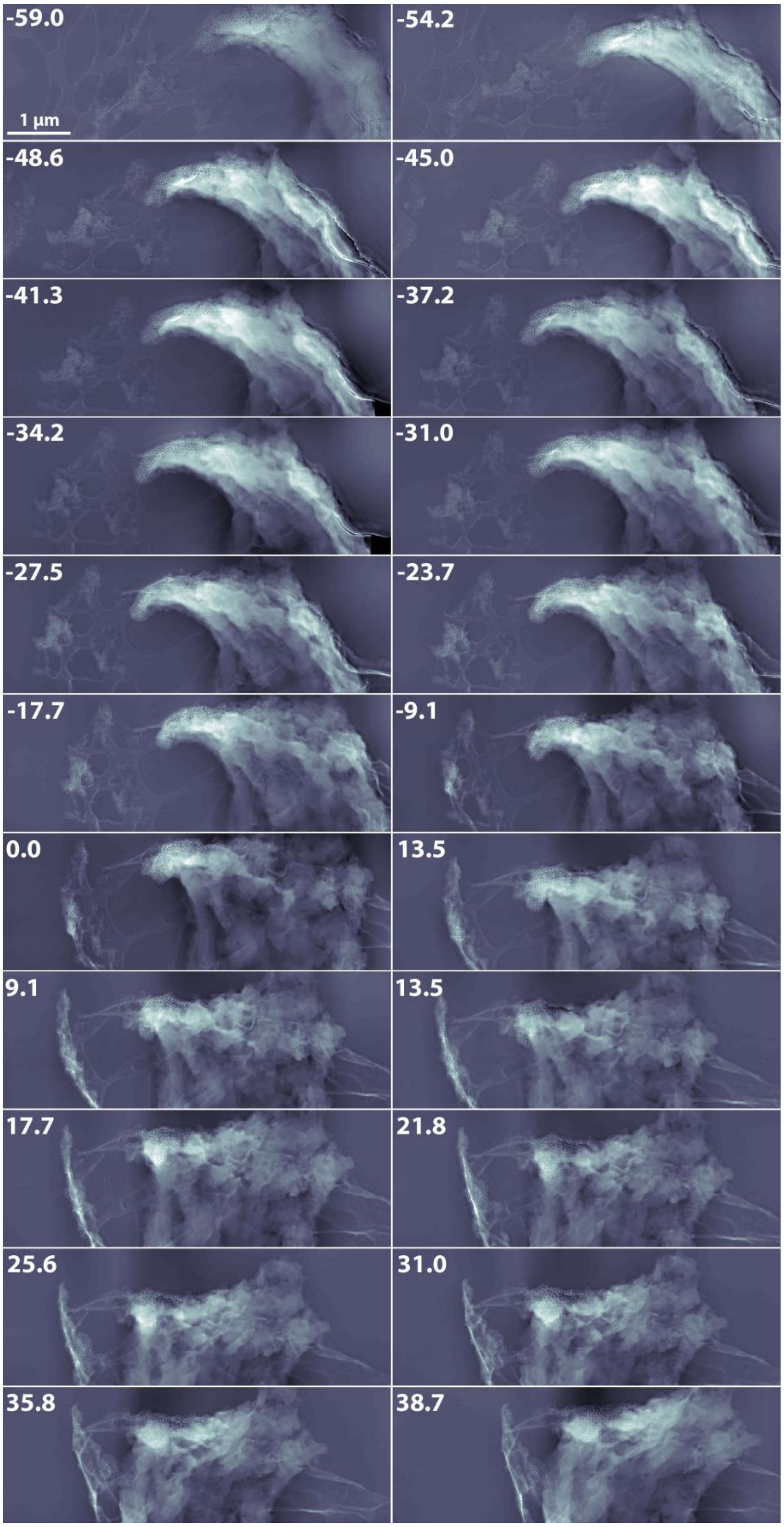

Supplementary Figure 6. Phase images of the ptychographic tomography tilt series ranging from -59° to +38.7° in equal slope increments.

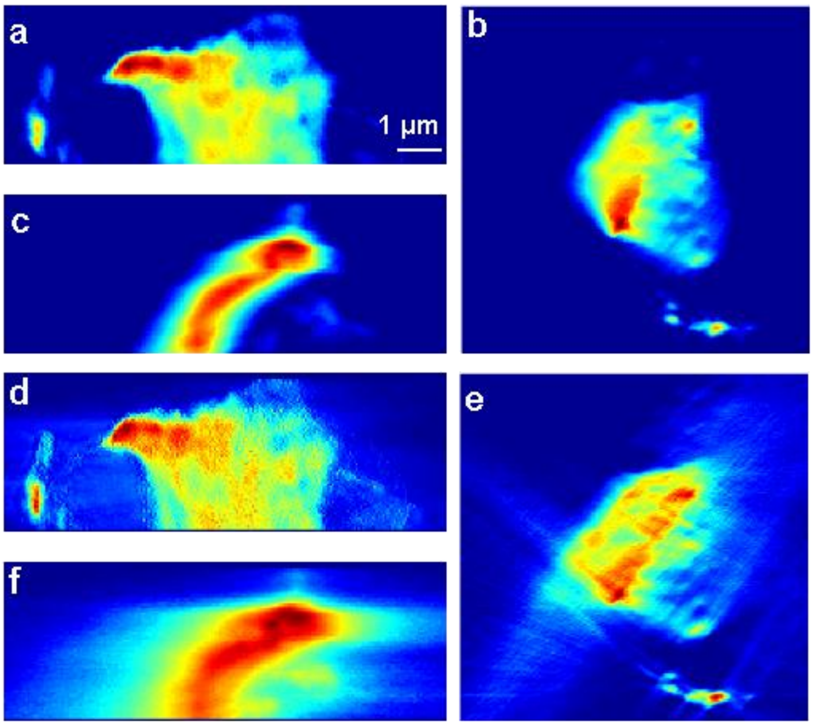

Supplementary Figure 7. Comparison of tomographic reconstruction methods using STXM projections. Three orthogonal projections are shown for GENFIRE in (**a**), (**b**), and (**c**), with corresponding views shown for filtered back projection in (**d**), (**e**), and (**f**).

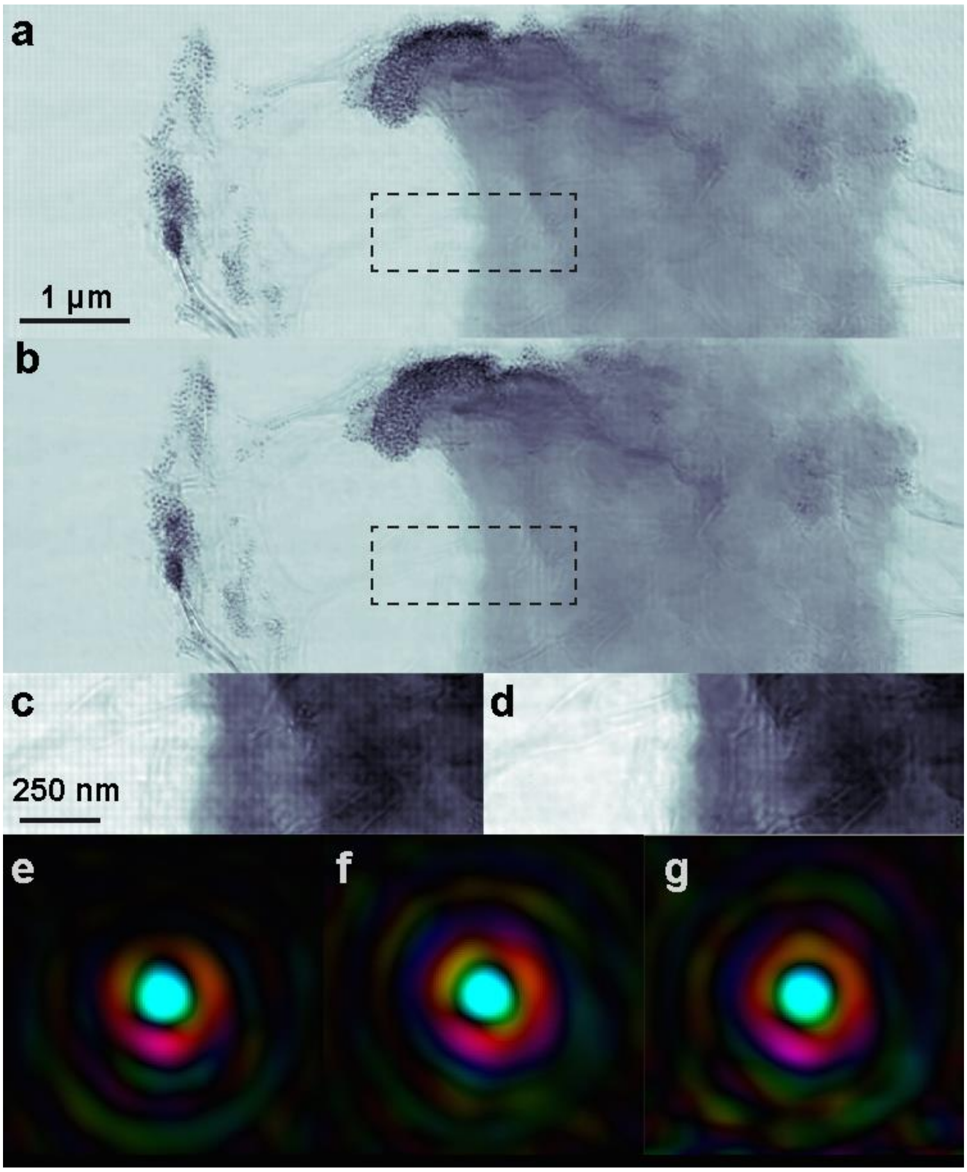

Supplementary Figure 8. Removal of reconstruction artifacts by relaxation of probe uniformity. (**a**) Initial reconstruction with a single probe showing several artifacts due to the regular grid and the OSA interfering with the probe. (**b**) Reconstruction from the same dataset using multiple probes with a strip wise averaging kernel, most of the more obvious artifacts have been removed. (**c**) Zoom in of the region highlighted in (**a**) showing a clear grid pathology. (**d**) Corresponding region from (**b**) showing that the gridding pathology has largely been removed. (**e** – **g**) Probes from different regions of the reconstruction, Left to right: first strip, middle strip, last strip.

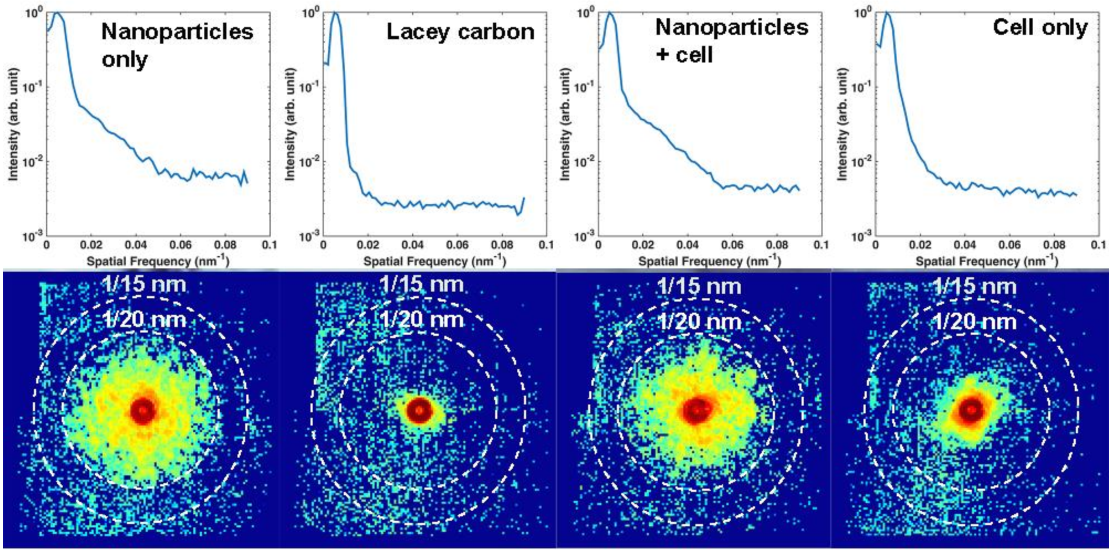

Supplementary Figure 9. Increase in scattering power in regions containing nanoparticles. Radial average profiles (top) and diffraction patterns (bottom) corresponding to the zoomed in regions shown in figure 4 demonstrating an increase in scattered photons at higher spatial frequency in the presence of nanoparticles.

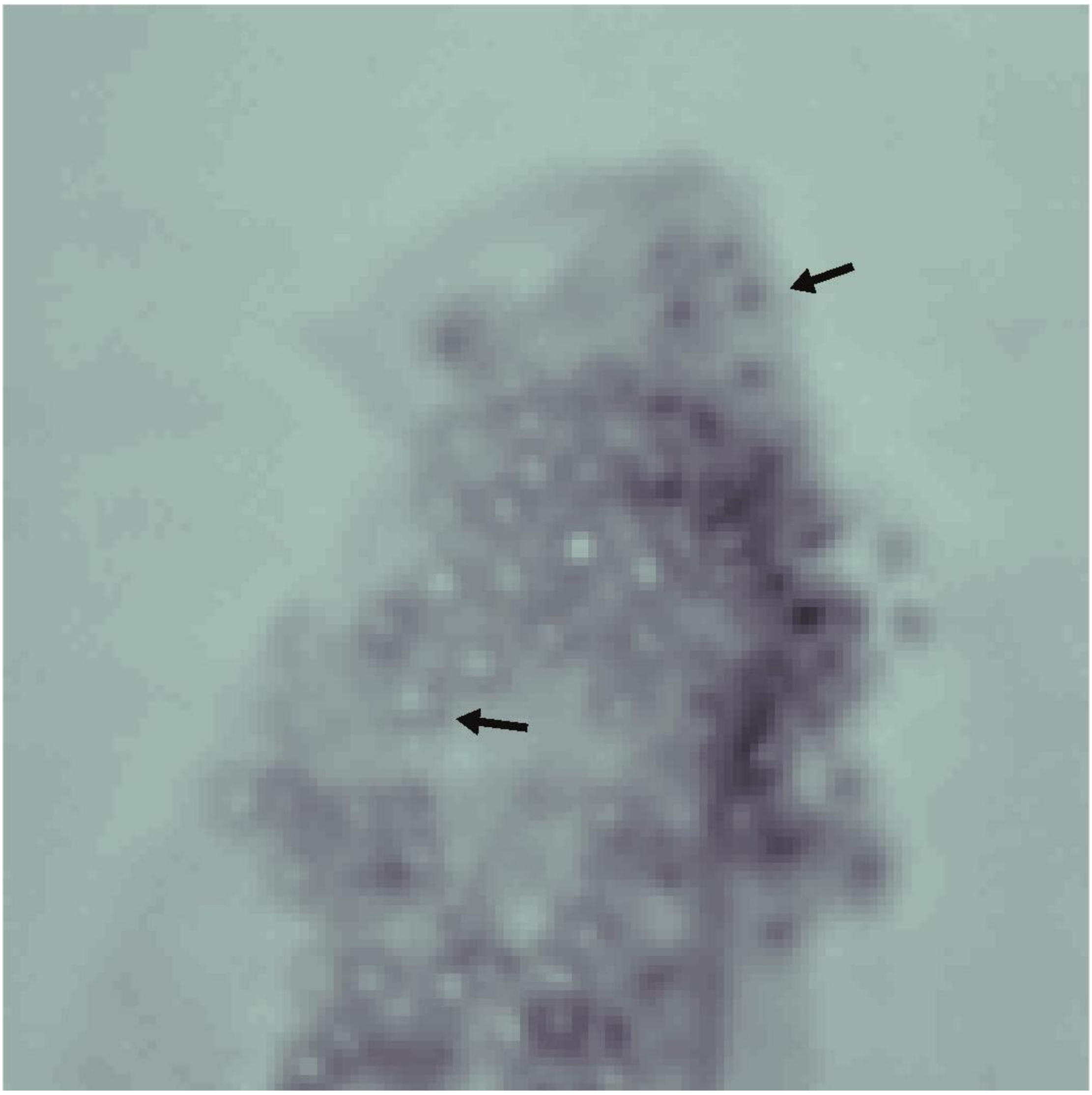

Supplementary Figure 10. Magnified view of nanoparticles from the phase of the ptychographic reconstruction. The two different oxidation states of nanoparticles (light and dark) are indicated by black arrows. The silica shell of the nanoparticles is also visible as a light grey annulus.

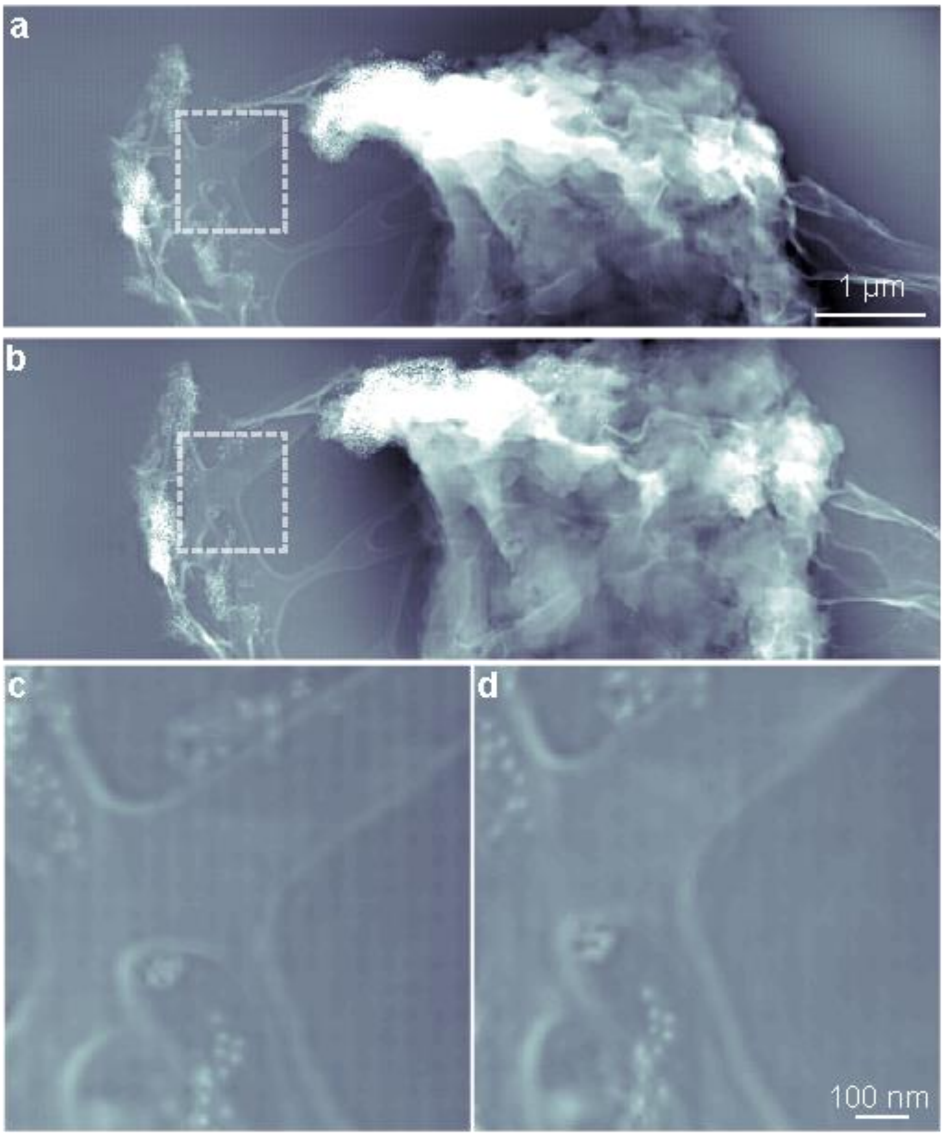

Supplementary Figure 11. Consistency of nanoparticle phase contrast across projections. Comparison of nanoparticles phase contrast between (**a**) -9.1° projections and (**b**) 0° projection. Corresponding zoomed regions are shown in (**c**) and (**d**), demonstrating that the phase contrast is consistent for each particle across multiple projections.

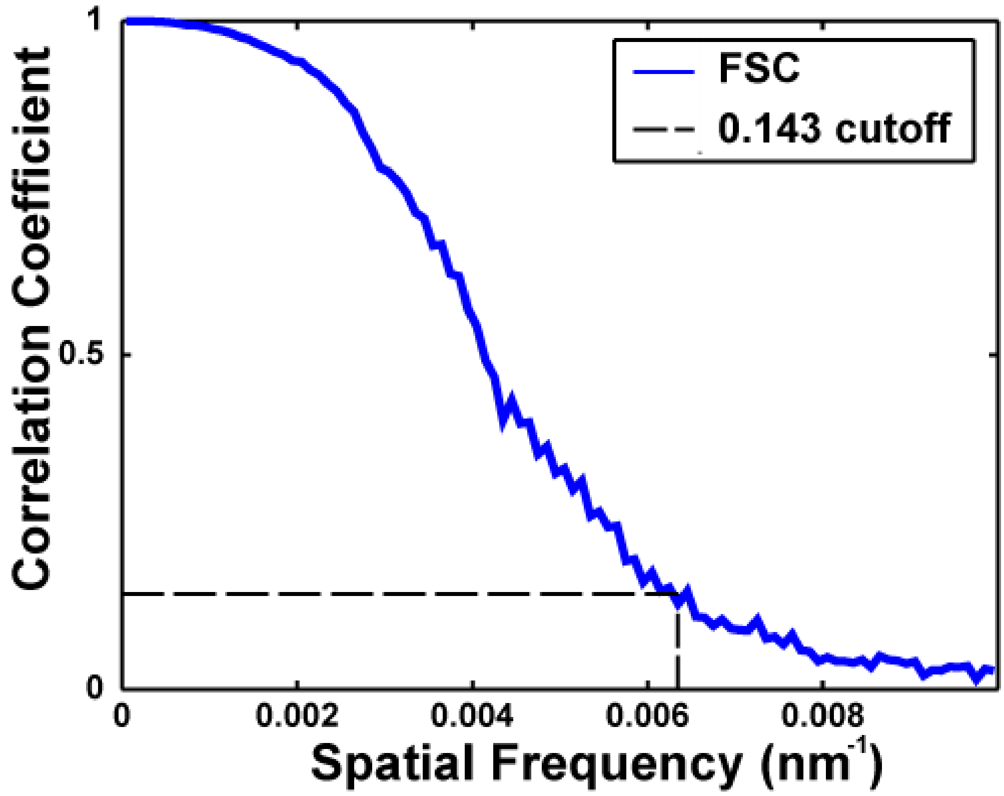

Supplementary Figure 12. Fourier Shell Correlation calculated from reconstructions of two half sets of the STXM tomography data. The dashed line indicates a resolution of 157 nm.

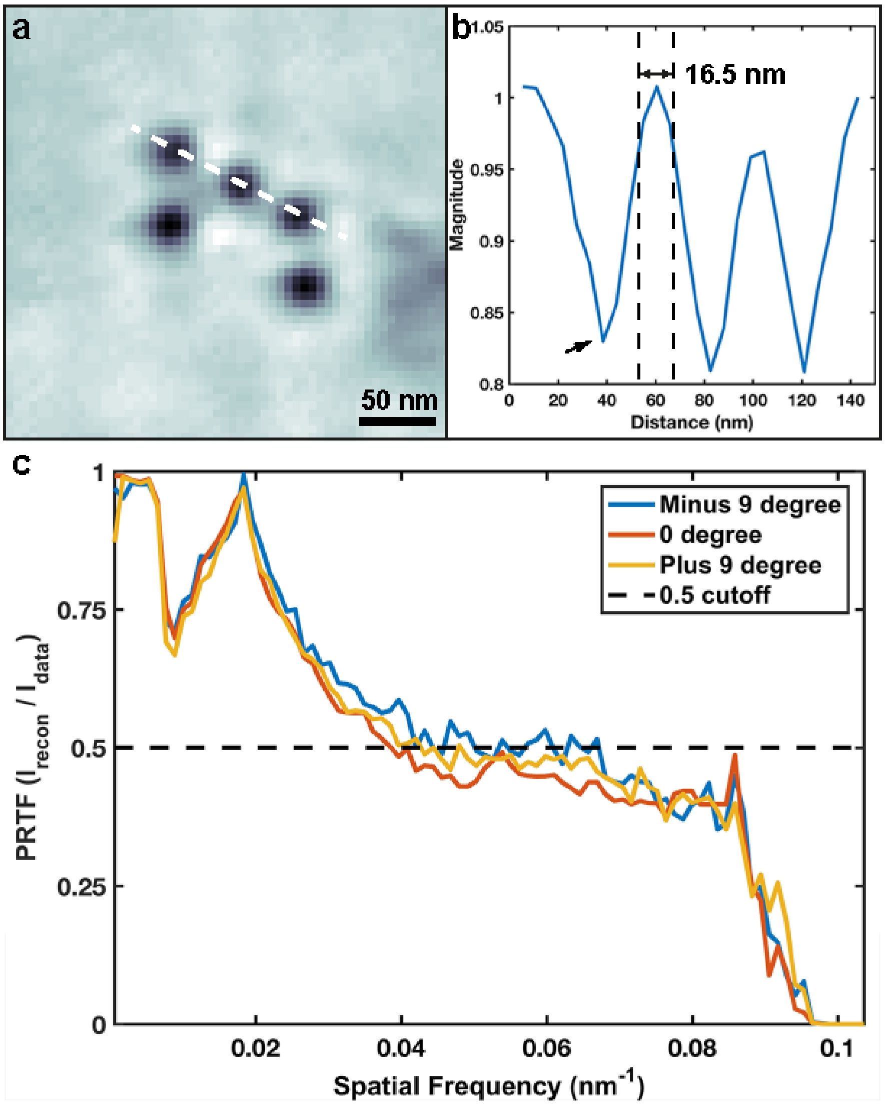

Supplementary Figure 13. Resolution estimates of ptychography reconstructions. (**a**) Zoom in of a region with well isolated iron oxide cores from the magnitude image of the -9.1° ptychography projection. (**b**) Line scan across the dashed white line in (**a**) showing clearly resolved edge-to-edge separation of 16.5 nm between individual nanoparticle cores. The black arrow indicates the center of the first core in (**a**). (**c**) Average phase retrieval transfer function (PRTF) calculated from all 7500 patterns in each dataset for the three principle projections used for further analysis. The cut-off at 0.5 shows that the resolution of the whole images lies somewhere between 25 nm and 15 nm.

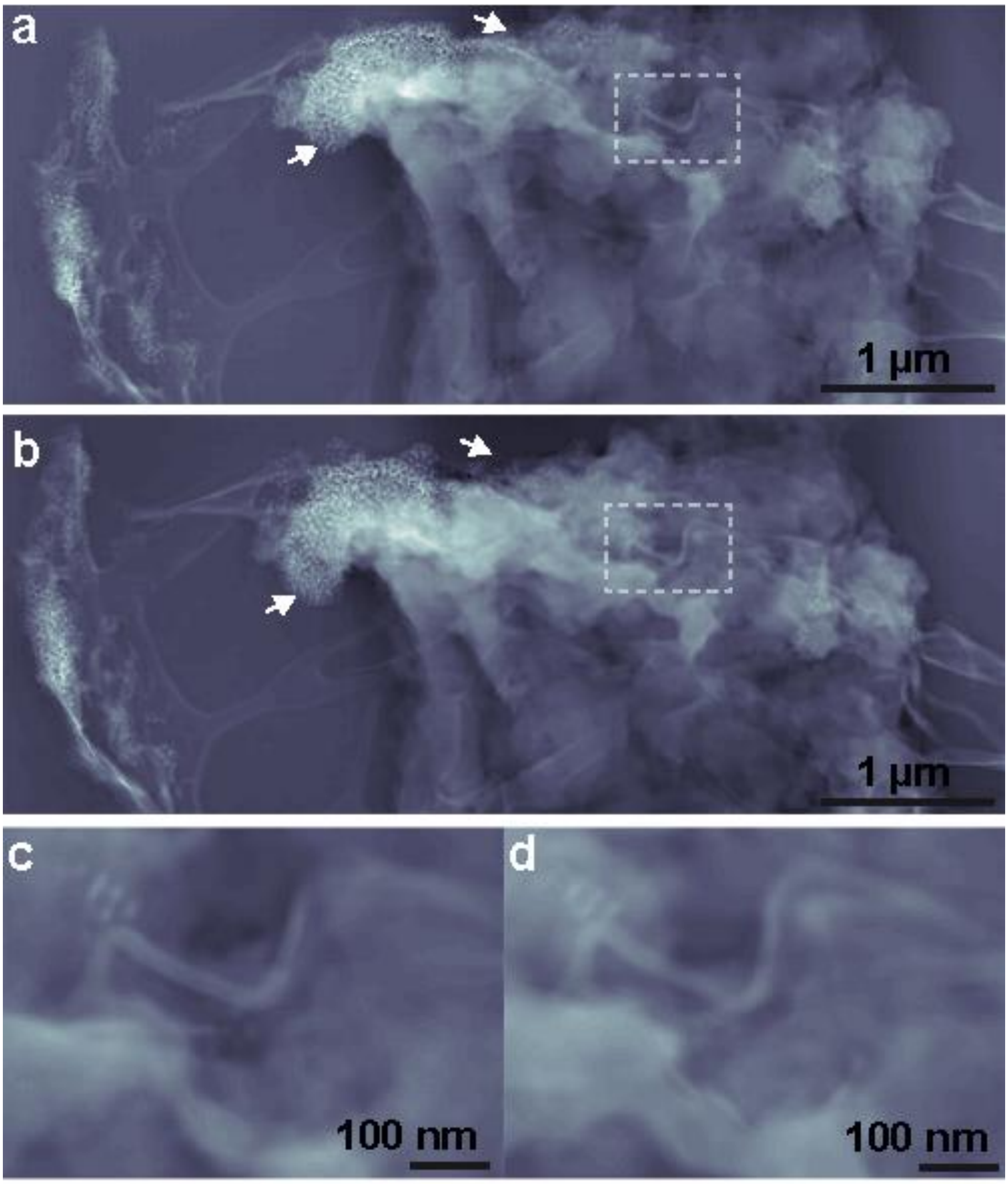

Supplementary Figure 14. Comparison of the 0° projection before and after tomography series acquisition. (**a**) Phase image of the zero degree projection before tomography. (**b**) Phase image of the zero degree projection after tomography. The two images show clear differences (highlighted by white arrows) mostly relating to an overall loss of mass of the cell due to radiation damage. (**c**) and (**d**) Zoom in of the highlighted region in (**a**) and (**b**) respectively demonstrating that fine features and nanoparticles remain largely unchanged during the full exposure time.
